# Double Arch Gingival Depigmentation With a Diode Laser for an Aesthetically Concerned Female: A Case Presentation

**DOI:** 10.7759/cureus.65912

**Published:** 2024-07-31

**Authors:** Shivani Thakre, Pavan Bajaj, Unnati Shirbhate, Sneha Dare

**Affiliations:** 1 Department of Periodontics, Sharad Pawar Dental College, Datta Meghe Institute of Higher Education and Research, Wardha, IND

**Keywords:** aesthetics, melanin, hyperpigmentation, laser, gingival depigmentation

## Abstract

The color of the gingiva is one of the gingival properties that affects soft tissue aesthetics and the general look of a smile. Demands for a pleasing smile that includes a healthy dentition and an aesthetically enhanced gingival component are rising. Melanocytes are the cells that are mostly found in the basal and suprabasal layers of the epithelium. Melanocytes deposit melanin on the gingiva that results in gingival depigmentation or hyperpigmentation of the gingiva. It's turning into a social stigma for which people are getting different treatments. Since the invention of laser (light amplification by stimulated emission of radiation), it has been employed extensively in both surgery and medicine. The use of a laser for depigmentation is rapid and easy.

This case study describes a laser-assisted gingival depigmentation procedure performed on a 25-year-old female patient who complained of dark-colored gingiva when she visited the periodontology department. It was decided to treat the maxillary and mandibular arches by depigmentation using a laser diode, with one week's interval between treatments. The patient was reviewed after one week and after three months of follow-up, the patient did not exhibit any repigmentation.

The primary reason for depigmentation is the patient's desire for a more attractive appearance. Individual preferences and clinical background should always be taken into consideration when selecting a technique because these elements produce more effective outcomes.

## Introduction

The quantity and function of melanocytes, the extent of the keratinized epithelium, and the level of circulation all affect an individual's oral mucosa color [1]. Patients who have a high smile line begin to consider the aesthetic aspects of their pigmentation. The color of healthy gingiva can vary from light pink to bluish-purple [2]. This pigmented gingiva is assumed to be composed of naturally occurring melanin pigments that contribute to the gingiva's endogenous coloration. Gingival depigmentation is the term for periodontal plastic surgery in which different procedures are used to remove or minimize gingival hyperpigmentation [3].

Dental aesthetic expectations are increasing for a pleasing look with healthy dentition and an aesthetically improved gingival component. The patient's request for aesthetic improvement serves as the first indication that the surgery needs to be performed. The patient's approval is required at each level before proceeding to the treatment [4]. The amount and number of blood vessels, the width of the epithelium, and the degree of keratinization are all reflected in the coral-pink gingival color. Conversely, gingival hyperpigmentation is directly caused by increased deposition of melanin in the basal and suprabasal layers of the oral epithelium [5].

The intraoral tissue that is most commonly pigmented is the gingiva. Microscopically, melanoblasts are typically found in the lamina propria's basal layers. The attached gingiva is the most frequent site (27.5%), with the papillary gingiva, marginal gingiva, and alveolar mucosa following in decreasing order of frequency [6]. All racial groups have gingival melanin pigmentation. It is thought that oral melanin pigmentation is multifactorial, resulting from a variety of physiological or pathological factors that may have localized or systemic origins. These include genetics, prolonged use of some medications, especially antimalarials and tricyclic antidepressants, and cigarette smoking [7].

Most people with gingival melanin hyperpigmentation do not have health issues; however, many may find their black gums unattractive. Patients who exhibit a high lip line or an extensive gingival show when smiling are more likely to have this issue. Gingival depigmentation removes or lessens gingival hyperpigmentation [4].

Gingival depigmentation can be achieved by a variety of techniques, including gingivectomy, gingival graft, electrosurgery, cryosurgery, radiosurgery, lasers, diamond bur abrasion, and chemical treatment (99% phenol and 95% alcohol) [8]. Since lasers have so many benefits, including easy handling, quick treatment times, good hemostasis, decontamination, and the absence of periodontal dressing, they have been cited by physicians as the most effective, compatible, and legitimate treatments among these methods. Wavelengths for diode lasers range from 600 to 980 nm. Diode lasers may effectively target tissues with darker pigmentation since these lasers are mostly absorbed by tissue chromophores, such as hemoglobin and melanin [9].

Advantages and disadvantages of laser

The following are some benefits of using a laser instead of a surgical procedure: non-invasive, bloodless surgery; instantaneous surgical field sterilization, minimal mechanical trauma, minimal bacteremia, minimal scarring and swelling following surgery, minimal pain following surgery, when compared to traditional procedures, it takes less chairside time, which leads to greater patient compliance. They can be employed with patients to help them cope with fear and anxiety. However, there are a few drawbacks to lasers, including their high cost, technique sensitivity, and requirement for protective eyewear on the part of the assistant, patient, and doctor [3].

This article shows how to use a laser diode for gingival depigmentation with a three-month follow-up.

## Case presentation

**Figure 1:**
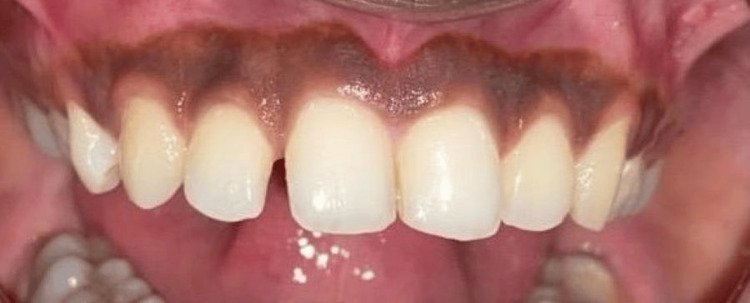
Pre-operative photograph with maxillary hyperpigmentation

**Figure 2:**
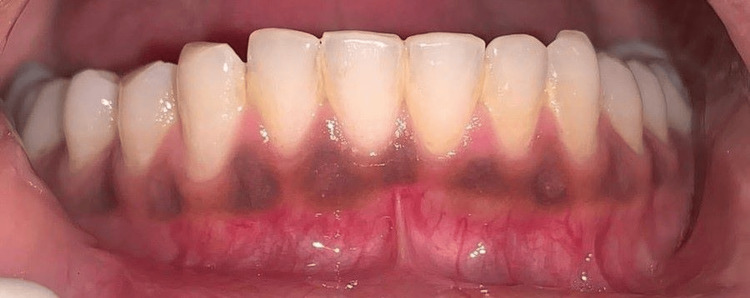
Pre-operative photograph with mandibular hyperpigmentation

A 25-year-old female patient reported to the Department of Periodontics and Implantology, complaining of poor aesthetics due to black gums with maxillary arch (Figure 1) and mandibular arch (Figure 2).

On oral examination, it was discovered that she had physiological melanin pigmentation, as evidenced by her darkly pigmented gingiva from childhood. The woman wished for any aesthetic therapy that would improve the appearance of her "black" colored gums. The patient maintained generally good and healthy dental hygiene.

**Figure 3:**
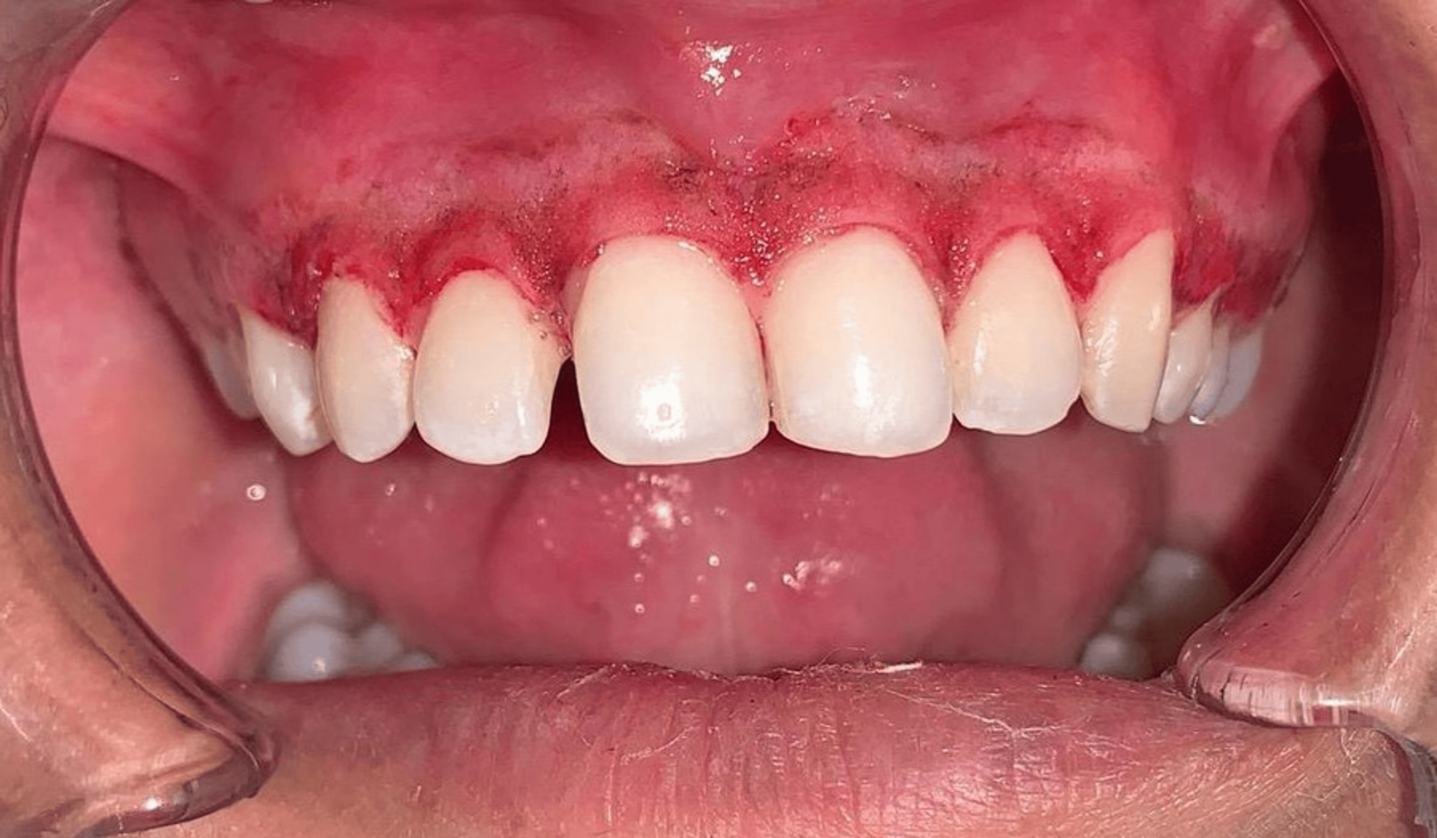
Post-operative maxillary arch just after the procedure

**Figure 4:**
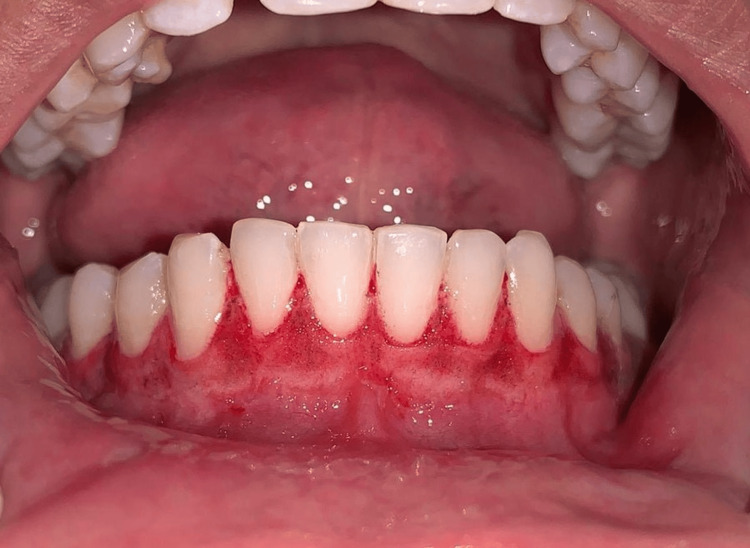
Post-operative mandibular arch just after the procedure

Scaling was performed during phase one therapy, and the patient opted for laser depigmentation from all the treatment choices provided. A diode laser with the following settings was used: wavelength of 940 nm, power of 1 W, fiber tip diameter of 300 μm, total energy of 180 J, and energy density of 4 J/cm^2^. The maxillary arch was treated first (Figure 3) and then the mandibular arch seven days later (Figure 4).

**Figure 5:**
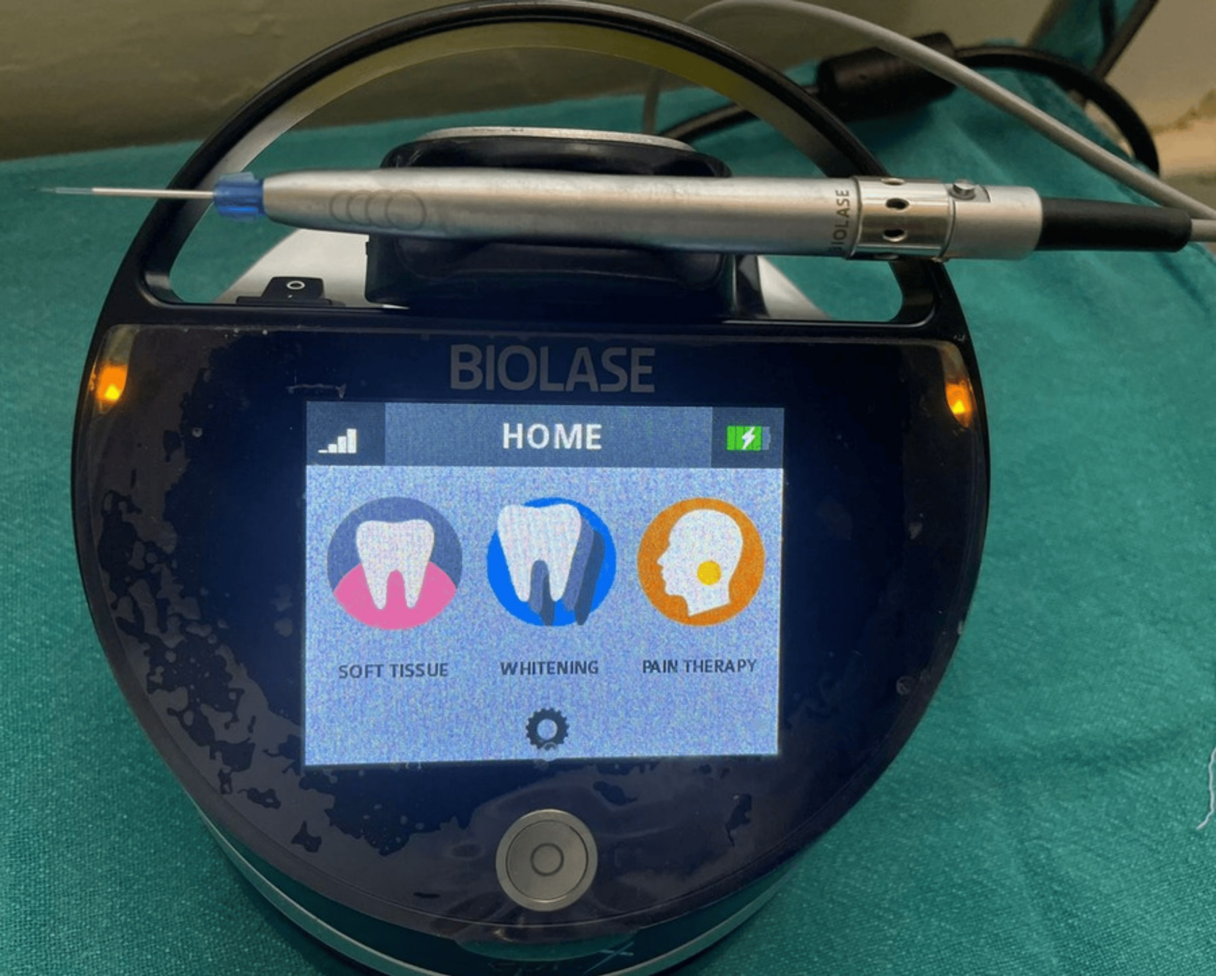
Laser used in the procedure

Figure 5 depicts the laser utilized.

To avoid overheating and tissue damage, the laser tip was moved in a controlled, sweeping manner across the pigmented areas rather than staying in one place for too long. The lighter tissue beneath the gum tissue was exposed when the outer layers were removed by the laser radiation. After that, saline-soaked gauze was used to clean the depigmented region.

**Figure 6:**
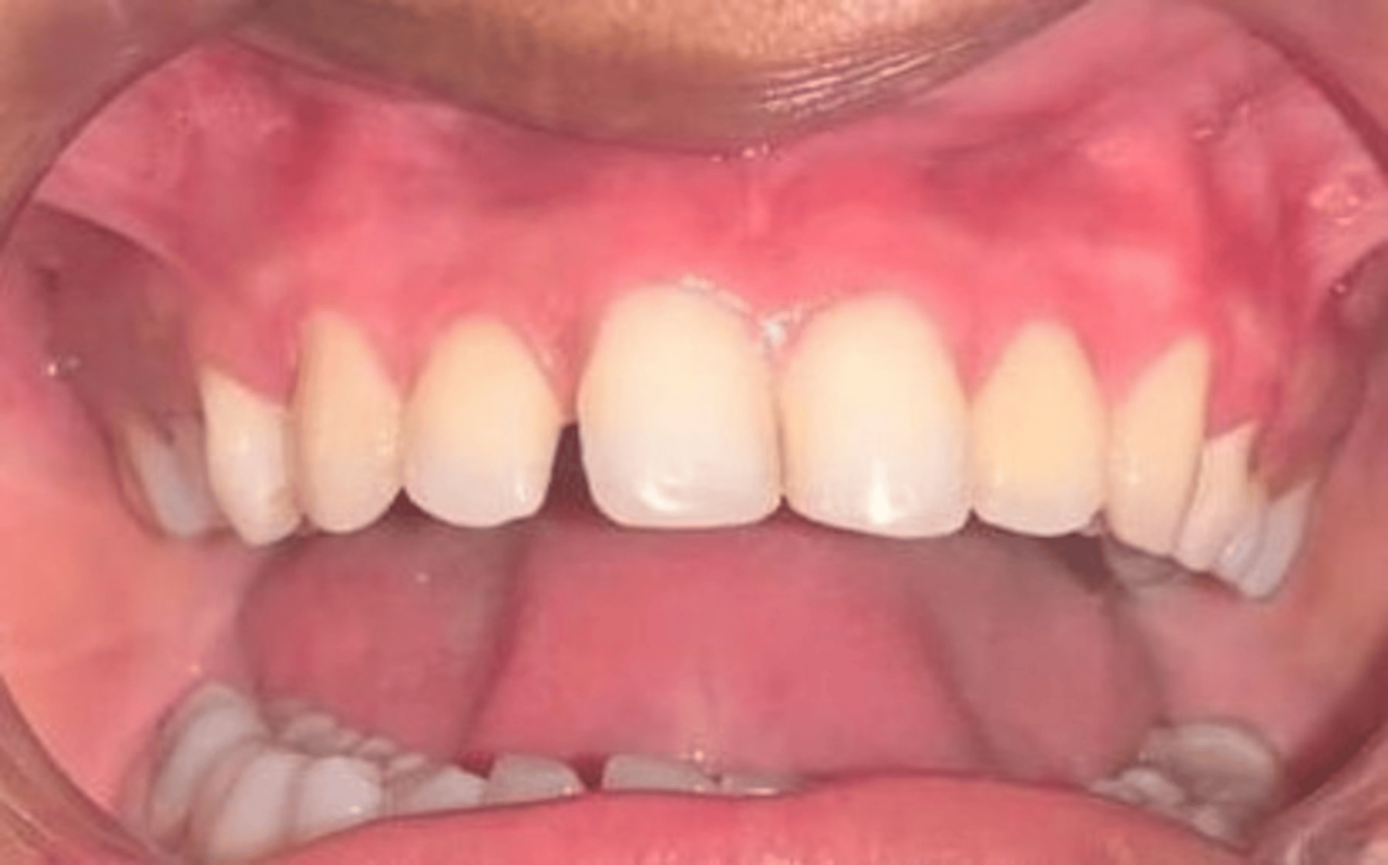
One week follow-up photograph of the maxillary arch

**Figure 7:**
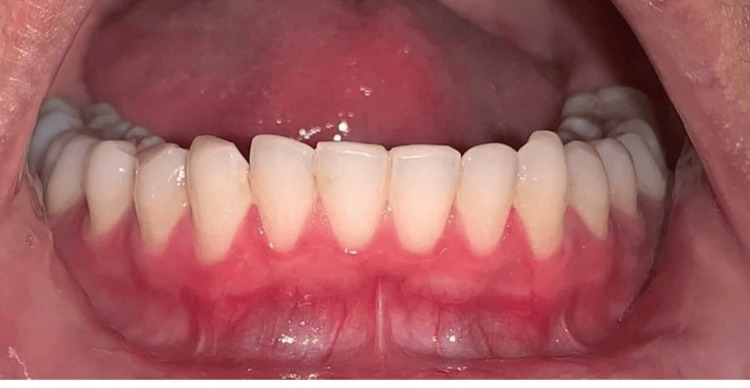
One week follow-up photograph of the mandibular arch

After one week, the patient was reviewed for maxillary arch (Figure 6) and mandibular arch (Figure 7).

**Figure 8:**
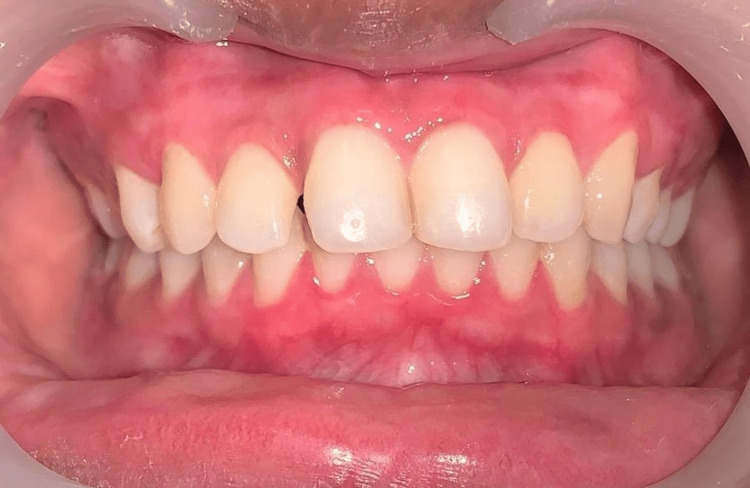
Post-operative photograph after a three-month follow-up

The patient reported minimal discomfort as the healing process progressed regularly. A three-month post-operative follow-up appeared normal since the gingiva was firm, pink, and healthy (Figure 8).

With no obvious repigmentation in this specific instance, it is possible to speculate that the technique used was visually satisfactory to the patients concerned. With no risks and bloodless surgery, the laser provides great results.

## Discussion

Melanin pigmentation is frequently caused by active melanocytes, which are mostly present in the basal layer of the oral epithelium and deposit melanin. Physiological pigmentation is most certainly genetically determined, irrespective of Dummet's proposal that the extent of pigmentation is primarily related to mechanical, chemical, and physical variables. It is possible to eliminate pigmentations for cosmetic purposes. For this purpose, many therapy techniques have been employed. The choice of a gingival depigmentation technique should take into account the patient's finances, preferences, and clinical experience. The need for aesthetic rehabilitation due to gingival melanin pigmentation is growing. For gingival depigmentation, several techniques have been proposed, including lasers, electrosurgery, cryosurgery, and scalpels [10].

Hassan et al. in 2022 [11] compared the effectiveness of using a laser and a scalpel to treat gingival hyperpigmentation. A 23-year-old female patient had diode laser treatment on her left side and scalpel treatment on her right. The authors concluded that there was no recurrence with any of the therapies they examined with a follow-up period of one month and that both techniques had similar healing outcomes. The laser used was a diode laser with a wavelength of 980 nm, although the burned layer acted as a bandage to control the flow, whereas following surgery, the bleeding area from the scalpel technique needed to be examined.

According to Vassoler et al. [12], gingival depigmentation with a high-power diode laser is a minimally invasive, effective treatment for melanin pigmentations in the oral cavity that produces acceptable aesthetic outcomes with little side effects.

Melanin depigmentation occurred in both the upper and lower quadrants. Several authors have conducted depigmentation solely in the upper quadrant due to patients' high smile lines and limited melanin pigmentation in the maxillary anterosuperior region [13-15].

This is evident from the literature review. Melanin uses a process called photothermolysis to transform the light energy it absorbs from the 980 nm laser into heat. According to the majority of authors, heat that reaches temperatures between 100 and 150°C promotes tissue vaporization, coagulation, and protein denaturation, which reduces trans- and post-operative bleeding [16].

## Conclusions

When treating individuals with gingival hyperpigmentation, a high-power diode laser proved to be a safe and efficient method that provided satisfactory results with little discomfort. There is a risk of relapse or repigmentation, depending on the method employed and the length of follow-up. This study suggests that using a diode laser is a safe and practical alternative approach, but additional research is needed to fully understand the efficacy of the current methods.
